# High incidence of newly diagnosed obstructive coronary artery disease regardless of chest pain detected on pre-procedural cardiac computed tomography angiography in patients undergoing atrial fibrillation ablation

**DOI:** 10.1097/MCA.0000000000001201

**Published:** 2022-11-19

**Authors:** Szilvia Herczeg, Judit Simon, Nándor Szegedi, Júlia Karády, Márton Kolossváry, Bálint Szilveszter, Bernadett Balogi, Vivien K. Nagy, Béla Merkely, Gábor Széplaki, Pál Maurovich-Horvat, László Gellér

**Affiliations:** a Cardiology Department, Heart and Vascular Center, Heart and Vascular Centre of Semmelweis University, Budapest, Hungary; b MTA-SE Cardiovascular Imaging Research Group, Heart and Vascular Center, Heart and Vascular Centre of Semmelweis University, Budapest, Hungary; c Medical Imaging Centre, Semmelweis University, Budapest, Hungary; d Cardiovascular Imaging Research Center, Massachusetts General Hospital-Harvard Medical School, Boston, Massachusetts, USA; e Atrial Fibrillation Institute, Mater Private Hospital, Dublin; f Royal College of Surgeons in Ireland, Dublin, Ireland

**Keywords:** ablation, atrial fibrillation, chest pain, coronary artery disease, coronary computed tomography angiography

## Abstract

Cardiac computed tomography (CT) is often performed before catheter ablation of atrial fibrillation to map atrial and pulmonary anatomy. Incident coronary artery disease (CAD) may also be diagnosed during cardiac CT angiography (CTA). Our aim was to assess whether coronary CTA might be able to identify a significant proportion of patients with obstructive CAD prior to their catheter ablation procedure event, even in asymptomatic patients.

**Methods:**

Consecutive patients undergoing pre-ablation coronary CTA for atrial fibrillation between 2013 and 2020 were retrospectively selected. Patients with previously diagnosed CAD were excluded. Obstructive CAD was defined as ≥50% luminal stenosis. We analyzed the relationship between obstructive CAD, any chest pain, and traditional risk factors.

**Results:**

Overall, 2321 patients [median age 63.0 (54.4–69.2), 1052/2321 (45.3%) female] underwent coronary CTA and 488/2321 (21.0%) were diagnosed with obstructive CAD. There was no difference regarding the rate of obstructive CAD in patients with any chest pain compared to patients without any chest pain [91/404 (22.5%) vs. 397/1917 (20.7%), *P* = 0.416, respectively). The following parameters were associated with obstructive CAD: age > 65 years [odds ratio (OR) = 2.51; 95% confidence interval (CI), 2.02–3.13; *P* < 0.001), male sex (OR = 1.59; 95% CI, 1.28–1.98; *P* < 0.001), hypertension (OR = 1.40; 95% CI, 1.08–1.81; *P* = 0.012), diabetes (OR = 1.50; 95% CI, 1.13–1.99; *P* = 0.006), dyslipidaemia (OR = 1.33; 95% CI, 1.07–1.66; *P* = 0.011) and history of smoking (OR = 1.34; 95% CI, 1.07–1.68; *P* = 0.011).

**Conclusions:**

The high prevalence of obstructive CAD even in patients without chest pain highlights the importance of additional coronary artery diagnostics in patients undergoing left atrial CTA awaiting catheter ablation for atrial fibrillation. These patients regardless of chest pain thus may require further risk modification to decrease their potential ischemic and thromboembolic risk.

## Introduction

Cardiac computed tomography (CT) angiography (CTA) before catheter ablation of atrial fibrillation provides important information on the anatomy of the left atrium and pulmonary veins to tailor the ablation strategy [1–4]. It has been argued, that cardiac CTA results in increased cumulative radiological exposure [5,6]. Nevertheless, if we gain more data from the cardiac CTA images, like incidental extracardiac alterations or information on coronary artery disease (CAD), patients may benefit from these new findings [6–8]. The endeavour for the holistic strategy of atrial fibrillation management – the Atrial fibrillation Better Care (ABC) management described in the 2021 European Society of Cardiology's Guidelines on cardiovascular disease prevention in clinical practice – lowers the risk of all-cause and cardiovascular death, first hospitalization and cardiovascular events as well [9]. This guideline underlines the importance of identification and management of risk factors and concomitant diseases, such as CAD [9]. Since symptoms suggesting ischemic heart disease, such as chest pain could be observed in atrial fibrillation patients also without CAD, predicting CAD in atrial fibrillation patients is often challenging [10]. However, an incidentally found obstructive CAD may require a new preventive strategy to decrease potential ischemic events. Moreover, the reclassification of the thrombotic risk stratification (CHA_2_DS_2_-VASc score) potentially affects the anticoagulant strategy of atrial fibrillation patients as well [8].

Multiple studies have shown that at least 10%, or in some publications even 70% of patients with atrial fibrillation have CAD [11–14]. Most of these referred publications used different definitions to determine the presence of CAD with lower spatial resolution CT scans assessing only a few hundred patients [8,11–14].

Our aim was to present a precise evaluation of CAD in a large cohort of consecutive atrial fibrillation patients undergoing high-spatial resolution coronary CTA before catheter ablation. We hypothesised that coronary CTA might be able to identify a significant proportion of patients with obstructive CAD prior to their catheter ablation procedure event, even in patients without symptoms.

## Materials and methods

### Study population

In our descriptive study, we screened all the consecutive patients undergoing coronary CTA imaging retrospectively between 2013 and 2020. Inclusion criteria were (1) coronary CTA indicated to tailor ablation strategy, (2) history of atrial fibrillation, (3) patients ≥18 years old. Exclusion criteria were the following: (1) previously known CAD (history of acute myocardial infarction and coronary revascularization, known CAD treated conservatively), (2) coronary CTA image quality non-diagnostic for coronary artery luminal stenosis assessment, (3) and repeated examinations of the same patient (in this case we involved the first good-quality pre-ablation scan).

Data regarding demographics, medical history and symptoms were collected from the questionnaires filled out by patients and other medical reports collected prior to coronary CTA. Overweight was defined as BMI above 25 kg/m^2^. Two groups were identified based on whether the patients had reported any type of chest pain or had no chest pain. Afterwards, we analyzed the relationship between these collected data and novel obstructive CAD diagnosed by coronary CTA.

All patients, who were enrolled in this study, gave written informed consent. The study protocol was reviewed and approved by the Local Research Ethics Committee (SE RKEB: 142/2019) and was in accordance with the Declarations of Helsinki.

### Cardiac computed tomography angiography – imaging

Cardiac CT examinations were performed on a 256-slice scanner (Brilliance iCT 256, Philips Healthcare, Best, The Netherlands) with prospective ECG-triggered axial acquisition mode. For coronary artery calcium score (CACS) a 120-kV tube voltage with a 30–50 mAs tube current was used. For coronary CTA, a 100–120 kV with a 200–300 mAs tube current depending on patient anthropometrics was set. Image acquisition was performed with 128 × 0.625 mm detector collimation, and 270 ms gantry rotation time. For heart rate control metoprolol was given, if necessary. In patients with a heart rate of <80 beats per minute (bpm), mid-diastolic triggering was chosen with 3–5% padding (73–83% of the R–R interval), and in those with ≥80 bpm, systolic triggering was applied (35–45% of the R–R interval). Iomoprol contrast material (Iomeron 400, Bracco Ltd, Milan, Italy) was used with 85–95 mL contrast material at a flow rate of 4.5–5.5 mL/s from antecubital vein access using a four-phasic protocol. Bolus tracking in the left atrium was applied to obtain proper scan timing. Sublingual nitroglycerin was given between the CACS and coronary CTA examinations. Non-contrast datasets were reconstructed with a slice thickness and increment of 2.5 mm, while coronary CTA datasets were reconstructed with 0.8 mm slice thickness and 0.4 mm increment.

### Image analysis

Evaluation of cardiac CTA images was performed offline by 22 cardiologist and radiologist experts. In case of any uncertainty, the images were reviewed and re-evaluated. Coronary artery status was analyzed using commercially available semi-automated software (HeartBeat-CS, Philips IntelliSpace Portal, Philips Healthcare). Stenosis severity was qualitatively reported according to the current Society of Cardiovascular Computed Tomography Guidelines: normal: absence of plaque and no luminal stenosis; minimal: 1–25% stenosis; mild: 25–49% stenosis; moderate: 50–69% stenosis; severe: 70–99% stenosis; occluded: 100% stenosis. In this current analysis, we defined obstructive CAD as a lesion with ≥50% stenosis [15]. In case of the presence of multiple lesions, the most severe stenosis was considered. Furthermore, total CACS was determined as well.

### Statistical analysis

Categorical variables were presented as numbers and percentages. As continuous variables showed non-normal distribution according to the Shapiro–Wilk test, they were reported as median and interquartile ranges. We examined the association of obstructive CAD and traditional cardiovascular risk factors with uni- and multivariable binary logistic regression analysis using the entry method. Comparison between patients with and without any chest pain was made by Mann–Whitney *U*-test for continuous variables, and Chi-square test for categorical parameters. Association between any chest pain and traditional cardiovascular risk factors was also analyzed with uni- and multivariable binary logistic regression analysis using the entry method. A two-tailed *P* value <0.05 was considered significant. The statistical analysis was done by IBM SPSS Statistics, Version 25 (IBM Corp., Armonk, New York, USA) software package.

## Results

### Studied population

**Fig. 1:**
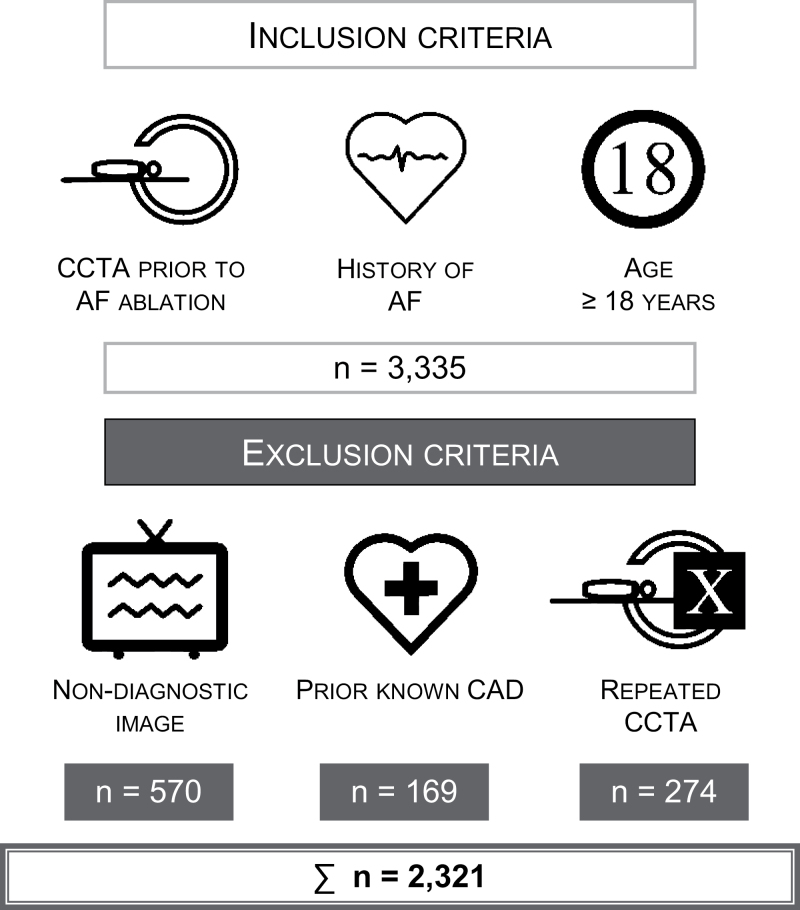
Illustration of our study population. CAD, coronary artery disease; CCTA, coronary CT angiography; CT, computed tomography.

We included 3335 pre-ablation coronary CTA examinations, from those 570 were excluded due to non-diagnostic images, and 169 patients because of previously known CAD. Additional 274 images were excluded, where multiple coronary CTA examinations were performed. In this case, the first good-quality image, only one for each patient, was included. Finally, 2321 patients with paroxysmal and persistent atrial fibrillation were included in our study population (Fig. 1).

The median age of the included patients was 63.0 (54.4–69.2) with the main characteristics shown in Table 1. The population was rather overweight: the median BMI was 28.7 (25.8–31.9) kg/m^2^. 68.1% (1580/2322) of the patients had hypertension, 34.6% (803/2321) dyslipidaemia and 31.2% (724/2321) history of smoking. 17.3% (401/2321) of the studied group reported having a positive family history of cardiovascular diseases. Furthermore, 13.5% (313/2321) of the involved ones suffered from diabetes. There were 404/2321 (17.4%) patients who mentioned any chest pain before the coronary CTA examination without previously known CAD.

**Table 1:** Characteristics of the studied population

Parameters	*n* = 2321	
Age (years)	63.0	(54.4–69.2)
Female sex	1052	(45.3%)
BMI (kg/m^2^)	28.7	(25.8–31.9)
Hypertension	1580	(68.1%)
Diabetes	313	(13.5%)
Dyslipidaemia	803	(34.6%)
History of smoking	724	(31.2%)
Positive family history of cardiovascular disease	401	(17.3%)
Peripheral vascular disease	108	(4.7%)
Prior stroke/TIA	126	(5.4%)
Any chest pain	404	(17.4%)

Continuous variables are presented as median and interquartile ranges and categorical variables as numbers and percentages.

TIA, transient ischemic attack.

### Comparison of patients with and without any chest pain

Patients with and without any chest pain were compared (Table 2). Patients with chest pain were older [64.8 (58.0–70.3) vs. 62.6 (53.5–68.9), *P* < 0.001], majority female [58.9% (238/404) vs 42.5% (814/1917), *P* < 0.001], having hypertension [72.3% (292/404) vs. 67.2% (1288/1917), *P* = 0.048], reporting positive family history for cardiovascular disease [24.3% (98/404) vs. 15.8% (303/1917), *P* < 0.001] and having peripheral vascular disease [7.7% (31/404) vs. 4.0% (77/1917), *P* < 0.001]. Obstructive CAD was in equal distribution in both groups [22.5 % (91/404) vs. 20.7% (397/1917), *P* = 0.416].

**Table 2:** Comparison of traditional cardiovascular risk factors of patients with and without any chest pain determined by Mann–Whitney *U*-test (number, percentage) and Chi-square test (median, interquartile range)

Parameters	Any chest pain (*n* = 404)	Without chest pain (*n* = 1917)	*P*
Age (years)^a^	64.8 (58.0–70.3)	63 (54–69)	<0.001
Female sex^b^	238 (58.9%)	814 (42.5%)	<0.001
BMI (kg/m^2^)^a^	28.7 (25.9–32.1)	28.7 (25.8–31.8)	0.679
Hypertension^b^	292 (72.3%)	1288 (67.2%)	0.048
Diabetes^b^	53 (13.1%)	260 (13.6%)	0.807
Dyslipidaemia^b^	145 (36.0%)	658 (34.4%)	0.534
History of smoking^b^	127 (31.7%)	597 (31.3%)	0.886
Positive family history of CV disease^b^	98 (24.3%)	303 (15.8%)	<0.001
Peripheral vascular disease^b^	31 (7.7%)	77 (4.0%)	0.001
Prior stroke/TIA^b^	25 (6.2%)	101 (5.3%)	0.460
Obstructive CAD^b^	91 (22.5%)	397 (20.7%)	0.416

CAD, coronary artery disease; CV, cardiovascular; TIA, transient ischemic attack.

a Mann–Whitney *U*-test.

b Chi-square test.

According to our multivariable analysis, factors associated with any chest pain were age >65 years (OR = 1.30; 95% CI, 1.03–1.64; *P* = 0.028), female sex (OR = 1.84; 95% CI, 1.47–2.30; *P* < 0.001), positive cardiovascular family history (OR = 1.70; 95% CI, 1.30–2.22; *P* < 0.001) and peripheral vascular disease (OR = 1.74; 95% CI, 1.11–2.75; *P* = 0.016). CAD was not associated with the symptom of chest pain (OR = 1.06; 95% CI, 0.80–1.39; *P* = 0.693) (Table 3).

**Table 3:** Factors associated with any chest pain reported as determined by uni- and multivariable analysis, using logistic regression

	Univariate OR (95% CI)	*P*	Multivariable OR (95% CI)	*P*
Age > 65 years	1.43 (1.15–1.77)	0.001	1.30 (1.03–1.64)	0.028
Female sex	1.94 (1.56–2.42)	<0.001	1.84 (1.47–2.30)	<0.001
BMI >25 kg/m^2^	0.97 (0.74–1.28)	0.837	0.96 (0.72–1.27)	0.758
Hypertension	1.27 (1.00–1.61)	0.048	1.20 (0.92–1.56)	0.170
Diabetes	0.96 (0.70–1.32)	0.807	0.81 (0.58–1.14)	0.234
Dyslipidaemia	1.07 (0.86–1.34)	0.535	0.93 (0.73–1.18)	0.563
History of smoking	1.02 (0.81–1.28)	0.886	0.97 (0.76–1.24)	0.822
Positive family history of CV disease	1.71 (1.31–2.21)	<0.001	1.70 (1.30–2.22)	<0.001
Peripheral vascular disease	2.00 (1.30–3.07)	0.002	1.74 (1.11–2.75)	0.016
Prior stroke/TIA	1.19 (0.76–1.87)	0.460	0.94 (0.59–1.51)	0.803
Obstructive CAD	1.11 (0.86–1.44)	0.416	1.06 (0.80–1.39)	0.693

CAD, coronary artery disease; CI, confidence interval; CV, cardiovascular; OR, odds ratio, TIA, transient ischemic attack.

### Coronary artery status of the studied population

**Fig. 2:**
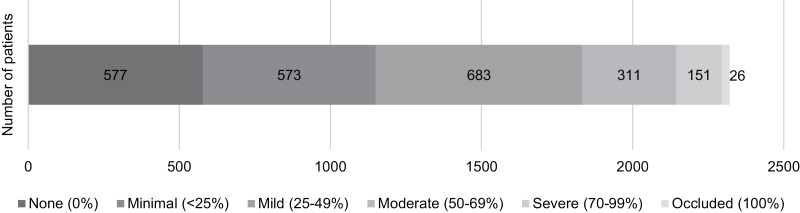
Distribution of stenosis severity (in case of multiple lesions, the highest observed severity is presented).

We analyzed the stenosis severity on all coronary artery segments. In our patient population, 577/2321 (24.9%) had no stenosis, in 573/2321 (24.7%) minimal, and in 683/2321 (29.4%) mild stenosis was observed. Moderate stenosis was found in 311/2321 (13.3%) of the patients, severe stenosis in 151/2321 (6.5%) and in 26/2321 (1.1%) occluded coronary arteries were diagnosed (Fig. 2). In total, 488 (21.0%) patients were diagnosed with obstructive CAD (≥50% luminal stenosis). The total CACS was median 17.8 [0.0–168.6].

Next, we determined different factors associated with prevalent obstructive CAD by uni- and multivariable tests (Table 4). In multivariable analysis age >65 years (OR = 2.51; 95% CI, 2.02–3.13; *P* < 0.001), male sex (OR = 1.59; 95% CI, 1.28–1.98; *P* < 0.001), hypertension (OR = 1.40; 95% CI, 1.08–1.81; *P* = 0.012), diabetes (OR = 1.50; 95% CI, 1.13–1.99; *P* = 0.006), dyslipidaemia (OR = 1.33; 95% CI, 1.07–1.66; *P* = 0.011) and smoking history (OR = 1.34; 95% CI, 1.07–1.68; *P* = 0.011) were defined as significant associated factors for obstructive CAD. Any chest pain was not associated with obstructive CAD (OR = 1.06; 95% CI, 0.81–1.40; *P* = 0.676).

**Table 4.** Factors associated with prevalent obstructive coronary artery disease as determined by uni- and multivariable analysis, using logistic regression

Parameters	Univariate OR (95% CI)	*P*	Multivariable OR (95% CI)	*P*
Age > 65 years	2.68 (2.19–3.30)	<0.001	2.51 (2.02–3.13)	<0.001
Female sex	0.78 (0.64–0.96)	0.018	0.63 (0.50–0.78)	<0.001
BMI >25 kg/m^2^	1.14 (0.88–1.48)	0.323	1.00 (0.75–1.32)	0.994
Hypertension	1.97 (1.56–2.50)	<0.001	1.40 (1.08–1.81)	0.012
Diabetes	2.07 (1.59–2.68)	<0.001	1.50 (1.13–1.99)	0.006
Dyslipidaemia	1.67 (1.36–2.04)	<0.001	1.33 (1.07–1.66)	0.011
History of smoking	1.23 (1.00–1.52)	0.054	1.34 (1.07–1.68)	0.011
Positive family history of CV disease	1.11 (0.85–1.43)	0.444	1.10 (0.83–1.45)	0.516
Peripheral vascular disease	2.04 (1.34–3.05)	<0.001	1.40 (0.90–2.18)	0.136
Prior stroke/TIA	1.67 (1.12–2.46)	0.010	1.39 (0.92–2.12)	0.121
Any chest pain	1.11 (0.86–1.44)	0.416	1.06 (0.81–1.40)	0.676

CI, confidence interval; CV, cardiovascular; OR, odds ratio; TIA, transient ischemic attack.

## Discussion

### Main findings

The main finding of our study is the relatively high incidence (21.0%) of coronary artery luminal stenosis in atrial fibrillation patients regardless of the reported symptoms. The incidence of obstructive CAD was similar in patients with and without any chest pain (22.5% vs. 20.5%). Factors associated with chest pain differed from the parameters associated with obstructive CAD.

### Relationship of chest pain and coronary artery disease in atrial fibrillation patients

There is still scarce evidence about the relationship between chest pain and atrial fibrillation. In a recent publication, Rottlander *et al*. conclude that there might be only a weak association between atrial fibrillation patients admitted to the hospital due to chest pain and relevant CAD [16]. Brown *et al*. presented, that 140 atrial fibrillation patients with chest pain syndromes had no increased risk for acute coronary syndrome compared to 683 matched control subjects (11.4% vs. 10.8%) [17]. Graf *et al*. studied 79 patients with typical chest pain (without reporting cardiac rhythm) and normal epicardial coronary arteries, where 65% had reduced coronary flow reserve [18]. They established as well, that clinical cardiac risk factor analysis may help in prediction of the individual probability of microvascular dysfunction [18].

In our cohort, chest pain was not related to obstructive CAD. Elderly, female patients, ones with a positive family history of cardiovascular disease, and patients suffering from peripheral vascular disease or hypertension were more likely to have chest pain. The difference between the associated factors for chest pain and obstructive CAD suggests that the reported chest pains are rather noncardiac or related to atrial fibrillation. These findings highlight the importance of coronary diagnostics in patients undergoing pre-ablation cardiac CTA, while patients without any symptoms could have hidden CAD as well.

### Obstructive coronary artery disease in the atrial fibrillation population

The comparison between the articles studying CAD in patients with atrial fibrillation prior to catheter ablation is sometimes confusing, since the definition of CAD is heterogeneous. Additional coronary evaluation is not a required protocol during pre-ablation cardiac CTA but CACS is an often available parameter on pre-ablation scans focusing on pulmonary vein morphology. CAC was visually detected at a very high, 70.1% among the 638 patients with atrial fibrillation in Dunleavy *et al*.’s publication [12]. Visual CACS estimation was also used in the study of Hillerson *et al*., where 59.6–63.5% CAC was found on 278 non-gated CT scans retrospectively [19]. Kornej *et al*. analyzed cardiac CTA data, where clinically relevant stenosis (≥75% luminal reduction) was observed in 27.7% of the 238 patients with atrial fibrillation with the exclusion of myocardial ischemia [20]. In multiple studies ≥ or >50% luminal stenosis of main coronary arteries was defined as CAD: Weijs *et al*. found underlying CAD in 49% of paroxysmal atrial fibrillation population including 390 patients [14], while Nucifora *et al*. reported similar, 41% obstructive CAD cases among 150 patients with atrial fibrillation [21]. In a recent study involving 94 patients, only 26% had obstructive CAD on coronary CTA [13]. In our current study, the most widely used definition of CAD, as 50% equal or more of luminal stenosis was applied. CAD was found with a high-resolution CT scanner even in one-fifth of our studied population involving more than 2000 patients.

Risk factors for CAD vary extensively. The CADAF-CT trial identified male sex, high number of co-existing coronary risk factors, elevated BNP levels, enlarged left atrial volume, high CACS, as independent risk factors of myocardial ischemia in 757 patients with atrial fibrillation [11,22]. Weijs *et al*. reported similar predictors for luminal stenosis while comparing 115 paroxysmal atrial fibrillation patients with ones with constant sinus rhythm [14]. Also, the presence of atrial fibrillation was named as a risk factor for obstructive CAD [14,21]. Besides male sex, age, diabetes, Framingham score and CHA_2_DS_2_-VASc score proved to be good predictors for CAD in a retrospective analysis of Rottlander *et al*. involving 566 paroxysmal or newly diagnosed atrial fibrillation patients [16]. However, according to Chen *et al*., CACS was also observed without any conventional cardiovascular risk factors in 58% of the studied 324 patients [8].

In our study, several parameters were associated with obstructive CAD in atrial fibrillation patients. Obstructive CAD is threefold more likely among patients >65 years old, and nearly twofold more likely in males and in patients with diabetes as well. Hypertension, dyslipidaemia and a history of smoking are also significant variables anticipating obstructive CAD. Interestingly, positive family history, and obesity did not show any correlation with CAD.

### Clinical impact of our study

Our recent investigation underlines the importance of defining the coronary artery status in atrial fibrillation patients. The determined associated factors and relatively high number of novel obstructive CAD suggest that it is worth extending the routine pre-ablation left atrial CT examination with characterization for coronary artery stenosis even for patients with no chest pain. For atrial fibrillation treatment, the holistic ‘ABC’ pathway is recommended by the European Society of Cardiology’s new prevention guideline [9]. The identification and management of concomitant diseases and cardiometabolic risk factors (‘C’) plays an equal role as the anticoagulation (‘A’) and better symptom (‘B’) management [9]. Since a newly diagnosed obstructive CAD could raise the ischemic risk of atrial fibrillation patients, lifestyle changes, closer clinical follow-up, altered medical treatment (e.g. statins, antiplatelets), further investigations (e.g. stress echocardiography, coronarography) could be needed based on other individual risk factors and chronic diseases [9].

A newly diagnosed obstructive CAD would reevaluate the CHA_2_DS_2_-VASc score as well, which potentially would modify the anticoagulation strategy in patients with atrial fibrillation. One observational retrospective review concluded that atrial fibrillation patients with obstructive CAD had a higher incidence of thromboembolic events (ischemic stroke and systemic thromboembolism) compared to controls adjusted for CHA_2_DS_2_-VASc score components and relevant variables [23]. In multiple studies, after finding incidental CAD by coronary CTA, CHA_2_DS_2_-VASc reclassification was needed in 20–50% of the involved atrial fibrillation patients [8,13,24]. Thus, anticoagulation treatment needed to be modified in 20% of the cohort according to the observation of Wang *et al*. [24].

### Limitations

The most important limitation is the retrospective nature of the study. Unfortunately, there was a significant lack of follow-up of the patients regarding the results of further investigations advised based on the coronary CTA image. Thus, we could not demonstrate our investigation’s ultimate clinical impact. Additionally, some patients were excluded from our study, due to the poor quality caused by tachyarrhythmia. It is possible, it added a selection bias to our study.

### Conclusions

The incidence of obstructive CAD (≥50% luminal stenosis) among patients awaiting ablation for atrial fibrillation is high (21.0%). Any kind of chest pain was not related to the incidence of obstructive CAD. Our study highlights the importance of CAD diagnostics in patients awaiting ablation regardless even in patients experiencing no chest pain. A newly diagnosed obstructive CAD could raise the ischemic and thromboembolic risk affecting further medical treatment strategies.

## Acknowledgements

The research was financed by the Thematic Excellence Programme (2020-4.1.1.-TKP2020) of the Ministry for Innovation and Technology in Hungary, within the framework of the Bioimaging program of Semmelweis University.

### Conflicts of interest

G.S. reports personal fees from Abbott, Bayer, Boston Scientific and Johnson and Johnson Medical outside the submitted work. The remaining authors have no conflicts of interest.
